# The Institutional Worker

**Published:** 1913-08-30

**Authors:** 


THE
### INSTITUTIONAL WORKER
Being a Special Supplement to "The Hospital"

### OUR BUREAU OF INFORMATION.

### Rules for Correspondents.

1. Every letter must be accompanied by the coupon to be cut from the back cover (inside page) of The Hospital, current issue, and must contain the name and address of the correspondent with pseudonym for publication if desired. Replies by post cannot be given, save under exceptional circumstances at the Editor's discretion.

2. Letters from Approved Homes in reply to special needs published in the Bureau should state terms and full particulars, and be sent prepaid under cover to the Editor of the Bureau with name written across coupon for identification.

3. Proprietors of Homes which have not yet been entered on the List of Approved Homes, but have spare accommodation likely to suit special needs, are invited to write for an application form for registration. The fee for registration, which includes two announcements of the Home in the Bureau and other privileges, is 10s.

4. All communications to be addressed to the Editor of The Hospital, 28 Southampton Street, Strand, London, W.C., and marked "Bureau of Information."

### INSTITUTIONAL FACTS AND FIGURES.

### The Hospital Linen Room.

The supervision of the linen supply is a department of institutional housekeeping which requires very careful organisation, for every article of linen must be strictly accounted for, and worn articles repaired or renewed. The centre of this department is the general linen room, or, as it is sometimes styled, the sewing room, for it combines a workroom for making up new material and repairing worn articles, as well as a store room for the general linen supply from which the stocks in use in the various departments are replenished from time to time.

Furniture and Fittings Required.

The room should be light and airy, it should be fitted with shelves and cupboards needed for the stock of linen, and provided with a work table, a sewing machine, and a gas stove for heating an iron. For the stocks of linen in daily use separate cupboards should be provided for each ward or department, although in a small hospital one cupboard or room may serve for all departments.

The Personnel.

It is the duty of the matron or housekeeper, according to the size of the institution, to superintend the work of the department. A sewing maid is usually placed in charge, and not infrequently members of the nursing staff are afforded opportunities of taking duty in the room in order that they may acquire a knowledge of this important branch of institutional management. Where the uniforms of the domestic and nursing staff are made on the premises, the services of a practical dressmaker may be necessary, but some matrons and housekeepers are experts in the work, and sacrifice much of their leisure in order to save expense.

Checking, Sorting, and Mending.

The chief business of the department is the establishment and maintenance of a proper system of checking, sorting, mending, and renewing linen. The sister or head of each ward or department should be held responsible for her stock, and be in a position to account for it from time to time. She should see that a return is prepared of all articles sent to the laundry—a book of duplicate forms should be provided for this purpose, so that a copy may be retained for reference. As soon as the linen is returned from the laundry she should compare it with the list, and before storing it in the cupboard eliminate all worn or torn articles and send them to the sewing room. Here they should be overhauled by the sewing maid or person in charge, who should set aside those which are past repair for the inspection of the matron or housekeeper, and repair and redistribute the remainder. The women patients not infrequently help to repair worn articles, but it is desirable that the articles given to them for repair should be issued from and returned to the sewing room, so that the matron may have an opportunity of inspecting them. The sewing maid or person in charge should account strictly for every piece that passes through her hands.

Taking the Inventory.

It is desirable that an inventory of linen in general use be taken twice a year, and it will be found more convenient to replenish the stocks by replacing discarded articles at these times than by doing so at irregular intervals. The number of articles eliminated and the additions made should be carefully recorded, and the account of reserve stock in the general linen room made up. All new articles should be marked with the name of the department, the date of issue, and the number issued. It is important that a careful account be kept of the discarded articles with a view to making use of them in the best possible way. Many of them can be cut down and used for other purposes or for patching, and old linen is always useful in the wards, and is often used in place of lint for patients requiring extensive dressings, as well as for covering splints.

The Linen Guild.

At many institutions the work of the department is considerably lightened by a linen league or guild, the members of which, in addition to making up a large proportion of the articles of bed and household linen and patients' garments, also provide the requisite material.—W. F. H.

### Comparison of Expenditure.

In dealing with the subject of account checking at a provincial hospital, "Institutional" suggests that it would be better to charge the cost of purchases as far as possible to a departmental stores account, and to enter each month in the analysis book the cost only of goods actually consumed or distributed, his idea being that a fairer comparison of figures could thus be made. It is obvious that the expenditure incurred during a certain period does not necessarily indicate the actual needs of the institution during that period, and it is certainly desirable in comparing costs that variations in the values of stocks in hand at the close of the periods compared should be taken into consideration. "Institutional" appears, however, to overlook the fact that his suggestion would seriously interfere with his system of accounting, for according to his statement his ledger is posted from the analysis book, and consequently if the suggestion were carried out it would neither be possible to draft an accurate account of expenditure nor to agree the tradesmen's statements with the personal ledger balances.

How to Solve the Difficulty.

The difficulty can quite easily be overcome by arranging with the storekeeper to draw up on the last day of each quarter a complete inventory of goods in stock. The inventory should state the cost of the articles arranged under headings in accordance with the Uniform classification. A book should be kept for the purpose, so arranged that values of stocks in hand at the close of corresponding quarters can be entered side by side, and the differences noted. The fluctuations can thus be readily taken into account when examining the comparative expenditure tables. In small hospitals it is not necessary for all departments to keep a stock account, for only small quantities of goods are stored, and these are issued almost immediately upon receipt. If proper methods be adopted in the store rooms and good judgment be used in replenishing supplies, the quantity of stock in hand at stated intervals does not fluctuate to any appreciable extent.—"Steward."

### QUESTIONS for AUGUST.

August is a holiday-making time, and therefore the questions we are publishing for this month are of such a nature as to be easy to answer without recourse to reference books. Question 2 touches on a branch of philanthropy which is coming greatly to the fore, and practical workers in this field are invited to give their experience which cannot fail to be of considerable help to the many beginners whom this work is attracting. Question 1 should appeal to the secretariat of any institution, and their views, with the reasons for such views, will be welcome.

**Any Use for an Addressograph ?**

**1. Is it or is it not an advantage for a hospital to possess an addressograph? Give reasons, and if an advantage give particulars of cost.**

**Mothers' Dinners Menus.**

**2. Give a week's menu for dinners for mothers in connection with Schools for Mothers, with the daily cost for 20 people, bearing in mind the need for nourishing food and for strict economy.**

RULES.

The following rules must be observed:—

1. Contributions must be written on one side of the paper. They must bear the name and address of the sender and be accompanied by coupon to be cut from the back cover (inside page) of the current issue of The Hospital. A pseudonym must be chosen if the name is not to be published.

2. They must be addressed to the Editor of The Hospital, 28 & 29 Southampton Street, Strand, London, W.C., so as to reach him by the last day of the month for which the questions are set, and must be marked in the left-hand corner "Facts and Figures."

**A minimum payment of Five Shillings will be made for each published answer.**

### Any Use for an Addressograph?

We publish hereunder a reply to the first question for the month, stated above:—

I am not an institutional secretary, but have had a fair amount of secretarial work one way and another, and at one time contemplated the purchase of an addressograph, but looking back over past experience, and asking the question as to the utility of such a machine for institutional purposes, I should certainly answer in the negative.

The addressograph is of the greatest possible use to a firm, say, who circularise their customers monthly, or to a co-operative society, with its thousands of members, but hospitals, unfortunately, have not, generally speaking, that number of subscribers, and, further, subscribers do not receive a communication from their secretary each month! There is but the annual report, the annual appeal it may be, and perhaps a special request of some kind; surely it is not economy to obtain an addressing machine to address letters three times a year, for it must be remembered the initial cost is considerable, and again, changes of address occur, and every change necessitates further expense.—"Clericus."

### THE EDITOR'S LETTER-BOX.

*THE SICK AND IN NEED.*

### Home Required for District Nurse.

We shall be glad to hear from any of our readers who can suggest some means of helping a district nurse who has completely broken down in health with bad heart disease, and has been unable to work for the past year. She is only a little over thirty years of age, but there is no prospect of her continuing her nursing career, and she is entirely without means. She is a good needlewoman, and can do light work of this kind when well enough; at present she is able to get about and help herself, but she is quite unfit for regular employment. What is really required is a Home where she can be received free of charge, but any suggestions or offers of assistance will be welcome. Could any of our readers support her candidature for admission to the Royal Hospital for Incurables, Putney, or for a pension from that institution? Replies should be addressed to "V. H. B.," 170.

### Home Required.

A nurse, who has been suffering from a prolonged illness and is now convalescent, seeks a Home where she can stay until strong enough to work again, and rest as much as possible in the open air. As her illness has already cost her a considerable sum, she cannot afford to pay much more than 25s. or 30s. per week. Approved Homes are invited to reply to "M. E. K.," 171.

### Case of Hemiplegia.

St. Peter's Memorial Home, Maybury Hill, Woking, which receives women suffering from illnesses requiring prolonged periods of skilled treatment and nursing, for a weekly payment of 10s. 6d., would possibly suit your sister who is recovering from hemiplegia. Application should be made to the Sister-in-Charge.—"C. E. J."

### Cripples' Homes.

Apply to the Superintendent, Dartmouth Home for Crippled Boys, Eastmoor House, Blackheath, S.E. Crippled boys between the ages of 8 and 13 years are received at this institution upon the payment of £15 a year, and the sum of £3 10s., for the purchase of an outfit, upon entering the Home. The boys receive a general education and industrial training, the latter comprising a course of instruction in tailoring and boot-making. At the National Industrial Home for Crippled Boys, Wright's Lane, Kensington, W., boys between 13½ and 17 years are received for a period of three years' training. The yearly payment required is £10, and a single payment of £6 16s. 4d. for outfit. Application for admission should be made to the Secretary. Inasmuch as the main object of these institutions is to equip the boys for earning their livelihood, those whose infirmities render them unfit for the necessary training are excluded.—"Almoner."

*EMPLOYMENT AND TRAINING.*

### The Medical Profession.

It would be quite impossible for you to qualify as a medical practitioner by studying in your spare time. The study of medicine demands the surrender of the student's entire time for a period of at least five years at a medical school. Full particulars of the medical schools of the United Kingdom, the cost of curricula and scholarships available, are published in The Hospital in the first week in September of each year, and we suggest that you should study these. If you care to give us details as to your education and circumstances we will consider your prospects of gratifying your desire and further advise you.—"E. C. H."

### Ship's Stewardess.

The position of ship's stewardess is often obtained by personal recommendation, but unless you know anyone who can help you in this way, you should apply to the shipping companies. Write to the Royal Mail Steam Packet Co., 18 Moorgate Street, E.C.; the Prince Line, 118 Fenchurch Street, E.C.; the White Star S.S. Company, Liverpool; the Union-Castle S.S. Co., Ltd., 3-4 Fenchurch Street, E.C.; and the Booth S.S. Co., Ltd., Tower Buildings, Water Street, Liverpool.—"B."

*ACKNOWLEDGMENTS.*

**V. H. B.**—We are appealing to our readers on behalf of the district nurse who is suffering from heart disease, and we trust that some assistance will be forthcoming. Meanwhile, as the case is eligible for relief by the Royal Hospital for Incurables, Putney, we suggest that steps be taken to secure her admission to that institution or an out-pension. The waiting period is usually long, though it depends upon the support obtainable. The average number of votes required for admission to the hospital is 1,400. Application should be made to the Secretary, 4 St. Paul's Churchyard, London.

**M. E. K.**—We are making it known in these columns that you require a convalescent home, and we will shortly place you in communication with some Homes which have been personally recommended to us by medical men and others. As regards rooms in the neighbourhood of Kensington, you will find comfortable accommodation at the Nurses' Club, 56 and 57 Kensington Gardens Square, W.

**Nurse P.**—We are glad to hear that you have already received an application for admission to your Home, and we trust that you will shortly fill your vacancy for a child.

**M. J. B.**—We fear that the only way to obtain a position such as you seek is to apply to a registry office or adververtise your requirements.

*Communications received from S. Sedgwick, "Romana," and L. Starey are being attended to.*

### Editor's Notices.

**Contributions:**

Contributions should be written, or preferably typed, on one side of the paper only, and all articles sent in are accepted upon the distinct understanding that they are forwarded to The Hospital only.

The Editor cannot undertake to return MSS. not used, but when a stamped directed envelope is enclosed the MSS. may be returned if a special request is made.

Accepted articles and paragraphs of news will be paid for after publication at the scale rate.

**Address:**

To prevent delay all contributions and letters on editorial business must be addressed exclusively to the Editor, "The Hospital," 29 Southampton Street, Strand, London, W.C. It is important that this regulation shall be strictly observed.

**Correspondence:**

Correspondence on all subjects is invited. The name and address of correspondents must be given as a guarantee of good faith, but not necessarily for publication.

**Special Articles:**

Special articles are invited, and questions, inquiries, and paragraphs upon all matters relating to the work, administration and management of general, special, mental, fever, cottage and convalescent hospitals, sanatoria, homes, institutions, societies and organisations for the treatment or care of the sick, injured and dependants of all classes. Every matter affecting the interests and welfare of the staff of all grades working in these institutions will receive special consideration.

**Administrative Medicine, Research and Items of News:**

Special terms will be paid for approved articles on subjects in this wide field by experts who have made some section of it their special study and interest. We wish to give prominence to every movement tending to advance accuracy of diagnosis, efficiency in treatment, and the development of modern methods to eradicate disease and promote the welfare of the suffering.

**A Bureau of Information:**

We invite inquiries and applications for information and help in providing for that numerous class of sufferers who require special care or house-room which they cannot obtain in their own homes or secure for themselves. We cannot, however, prescribe, or recommend practitioners

**Books for Review:**

Publishers are particularly requested to send advance proofs of any new books of importance whenever possible, as the Editor has made arrangements to publish immediate reviews on a new plan.

**Photographs, Plans, Blocks, and Illustrations:**

It is requested that wherever possible MSS. may be accompanied by illustrations in any of the above forms. The name of the sender and of the article to which it belongs should be written on the back of each photograph, drawing, or block for purposes of identification.

**Local Papers:**

Newspapers containing reports or news paragraphs should be marked and addressed to the Sub-Editor.

**The Coupon System:**

A coupon will be found at the bottom of the third inside page of the cover each week. This coupon must be attached to every question or inquiry to which an answer is desired in The Hospital.

### It is to Your Interest to send for the Booklet containing full particulars of The Hospital Bureau of Information.

It is the purpose of this Department to become a practical help to *every* Institutional Worker, Health Visitor, District Nurse, and all associated with the Prevention of Disease, Care of the Sick, and the Advancement of National Health.

Years of experience in Institutional Affairs and the Medical Health and Sanitary Services are placed at the disposal of every reader of The Hospital requiring assistance entirely without charge.

The want of accurate information on the various phases of Institutional Work has been the stumbling-block in the past to many individual workers, but with the inauguration of The Hospital Bureau of Information this need be no longer a bar to progress.

A knowledge of the scope and work of the Bureau is a matter that deeply concerns your Professional career, and it is to your interest to obtain at once details of this new development of The Hospital, the Journal of the Institutional Worker.

All letters in regard to the Bureau must be addressed to The Hospital Bureau of Information, The Hospital Building, 28-29 Southampton Street, Strand, London, W.C.

### Manager's Notices.

**Letters relating to the publication, sale, and advertising departments of "The Hospital" must be addressed to The Manager, "The Hospital," The Hospital Building, 28 and 29 Southampton Street, London, W.C.**

**Advertisements:**

To ensure insertion, all advertisements for The Hospital must reach the Manager not later than Wednesday morning in each week. For scale of charges see pages v and 4.

**Remittances:**

**It is especially requested that remittances be made by Cheque or Postal Order, and in halfpenny stamps only when the amount is under sixpence.**

**Subscriptions** may begin at any time, and are payable in advance. Cheques and Post-Office Orders should be crossed London County and Westminster Bank, Covent Garden Branch, and made payable to the Manager of The Hospital.

Rates of Subscription (including postage).

| *United Kingdom.* | | *Foreign and Colonial.* | |
|---|---|---|---|
| Three Months ... | ... 2s. 0d. | Three Months ... | ... 2s. 6d. |
| Six Months ... | ... 4s. 0d. | Six Months ... | ... 5s. 0d. |
| Twelve Months ... | ... 6s. 6d. | Twelve Months ... | ... 8s. 8d. |

*SUBSCRIPTION FORM.*

*Please send me* The Hospital *for...............months, commencing with your issue of.............................., for which please find enclosed.............................*

*Name*.....................................................

*Address* ..................................................
..................................................

*Date*.....................................

*To* ..................................... *Newsagent.*

Or to

*The Manager,*
The Hospital,
The Hospital Building,
28/29 Southampton Street,
Strand, London, W.C.

By Post **5/4**

**It is possible to secure "the most up-to-date and Complete Guide to Nursing that has yet been written."**

*Vide THE NURSING MIRROR.*

### A HANDBOOK FOR NURSES

**With Examination Questions based upon the contents of the Chapters.**

**By J. K. WATSON, M.D.Edin., C.M.**

**5th Edition, Entirely Revised, and Enlarged to 544 pages**

The title of this book very inadequately describes its scope. It would be more accurately entitled "*THE* Handbook for Nurses," since the information it contains renders it of real and genuine service to the modern Nurse. The fact that **31,500** copies of former editions have been sold is indisputable evidence of its value. The work forms practically an Encyclopædia of Medical, Surgical and Monthly Nursing, and there are few, if any, Nursing details that do not find representation in its pages. If you have not seen the New Edition we would strongly advise you to take the earliest opportunity of doing so.

It is obtainable of all Booksellers, or direct from . . . .

**THE NURSES' PUBLISHERS,**

**28/29 SOUTHAMPTON STREET, STRAND - LONDON, W.C.**

NEARLY

**400 NEW WORDS**

have been added to the

**NEW EDITION**

of

### The Nurse's Pronouncing Dictionary

**OF MEDICAL TERMS AND NURSING TREATMENT**

Compiled by **HONNOR MORTEN.**

Eighth Edition, thoroughly Revised and much Enlarged.

Edited by **MARY I. BURDETT.**

The new issue of this well-known Dictionary is the **most complete work** of the kind published. It has been completely revised and some hundreds of new words have been added, consequently the utmost reliance can be placed in the information it contains.

To be up-to-date you should GET A COPY TO-DAY.

**Price, in cloth, 2/- post free; in leather, 2/6 net, by post 2/8.**

*Of all Booksellers, or of—*

**The Scientific Press, Ld.**

(*The Nurses' Publishers*),

**28/29 Southampton Street, STRAND, LONDON, W.C.**

